# Technoeconomic Analysis of Microwave-Assisted Dry Reforming Integrated with Chemical Looping for Production of Methanol

**DOI:** 10.1021/acs.iecr.5c00543

**Published:** 2025-06-07

**Authors:** Omar Almaraz, Jarrett Riley, Srinivas Palanki

**Affiliations:** † Department of Chemical and Biomedical Engineering, 5631 West Virginia University, Morgantown, West Virginia 26506, United States; ‡ National Energy Technologies Laboratory, 3610 Collins Ferry Road, Morgantown, West Virginia 25607, United States

## Abstract

Methanol is a key component in producing formaldehyde, acetic acid, and methyl *tert*-butyl ether (MTBE) and supports a wide array of industries, including plastics, textiles, and automotive. It also plays a growing role in renewable energy solutions. However, the conventional production of methanol involves steam reforming of methane, which is very energy-intensive and produces significant quantities of the greenhouse gas carbon dioxide. In this research, a chemical looping scheme is combined with dry reforming of natural gas in a novel microwave reactor to produce an industrial quantity of methanol. A heat exchanger network is developed to substantially reduce hot and cold utility usage. The effect of the cost of purchasing carbon dioxide from an external source for dry reforming, the capital cost of the microwave reactor, and the cost of electricity on the net present value is analyzed. Technoeconomic comparison with the conventional industrial process that produces methanol via steam reforming of methane indicates that the chemical looping generates a significant positive net present value along with a substantial reduction in carbon dioxide emissions while producing methanol significantly below the U.S. Department of Energy’s goal of $800/ton.

## Graphical abstract

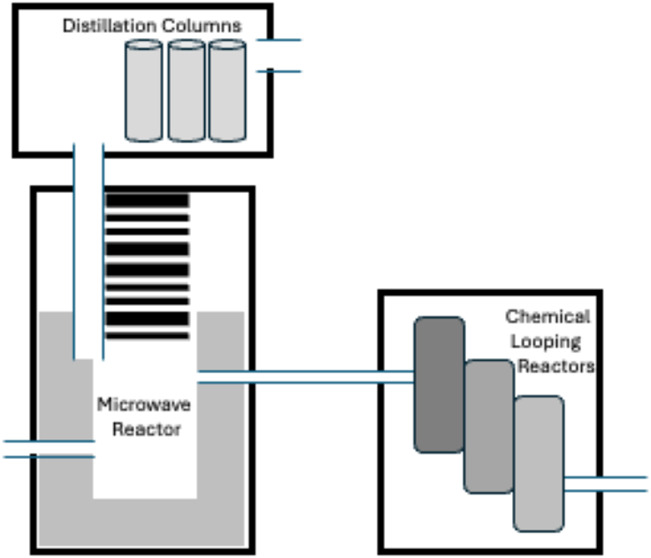

## 1 Introduction

Methanol is a versatile chemical widely used in industry as a feedstock and solvent.^1^ Historically, it was first isolated in the 17th century by pyrolysis of wood, earning it the name “wood alcohol.” However, its industrial production began in the early 20th century, with the development of the catalytic synthesis process by BASF in 1923. This method involved reacting carbon monoxide and hydrogen under high pressure using a zinc–chromium catalyst.^2^ Today, methanol production primarily utilizes natural gas as the feedstock, where methane undergoes steam reforming to produce syngas (a mixture of carbon monoxide, carbon dioxide, and hydrogen), which is then catalytically converted into methanol. This modern process has largely replaced older methods.^3^

Methanol serves as a building block for various chemicals, including formaldehyde, acetic acid, and methyl tertiary-butyl ether (MTBE), used in resins, plastics, and adhesives and as an octane booster in gasoline. It is also gaining prominence as a clean-burning fuel and a component in biodiesel production. Methanol-to-olefins (MTO) technology has further expanded its applications, converting methanol into ethylene and propylene, essential for producing plastics.^3^ Major manufacturers of methanol include Natgasoline, Methanex, SABIC, BASF, and PetroChina.

The current industrial production of methanol (also known as “gray methanol”) is energy-intensive, relying on fossil fuels, which contributes to greenhouse gas emissions. Steam reforming of natural gas, the most common method, generates significant carbon dioxide as a byproduct.^4^ However, efforts are underway to reduce the carbon footprint of methanol production through renewable energy sources and carbon capture technologies. Some manufacturers are exploring “green methanol” production using renewable hydrogen and captured carbon dioxide to lower emissions.^5^ While the shift toward sustainable methanol production is promising, scaling these technologies remains a critical challenge for the industry. The cost of methanol produced from green hydrogen and carbon dioxide strongly depends on the cost of green hydrogen and carbon dioxide as well as the cost of generating electricity from renewable sources. According to the International Renewable Energy Agency (IRENA), the cost of green methanol is estimated to be between $800–$1600/metric ton, whereas the cost of gray methanol made from steam reforming is about $250/metric ton.^6^ The United States Department of Energy’s Office of Fossil Energy and Carbon Management has the current goal of evaluating green methanol production technologies that result in a methanol cost below $800/ton.^7^ While incentives introduced by the Inflation Reduction Act in the U.S. can eventually spur research to reduce the cost of both green H_2_ and carbon dioxide captured from flue gas and make green methanol production cost comparable to gray methanol, it is important to consider alternatives that are cheaper than the current approaches to green methanol.

In this work, a novel microwave-assisted methanol process integrated with chemical looping is analyzed. This process has the following characteristics that are different from the conventional industrial process for producing methanol: (1) a novel multitube reactor that catalytically converts methane and carbon dioxide to syngas via dry reforming, (2) the use of microwave heating technology to reduce energy requirement and carbon dioxide emissions that result from burning hydrocarbons for process heat, and (3) an integrated chemical looping scheme that converts methane to a pure stream of hydrogen necessary for the methanol reactor while recycling the pure stream of carbon dioxide to the dry reforming reactor. The economic feasibility of this process is tested via simulation after the application of heat integration tools. This novel process is compared with the current industrial process of Natgasoline LLC.

## 2 Reaction Chemistry and the Development of Conceptual Flow Diagrams

Figure 1 shows a schematic of the current industrial process for making methanol at Natgasoline LLC.^8^ In this process, a stream of natural gas reacts with steam to produce syngas, which is further converted to methanol. The steam reforming reaction can be written as

1 $$CH_{4}+H_{2}O\to \mathrm{CO}+{3H}_{2}$$

**Figure 1:**
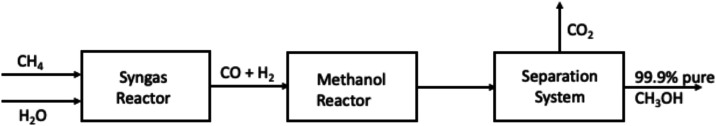
Current industrial process for methanol production.

Some of the carbon monoxide reacts further with steam to form carbon dioxide via the water–gas shift reaction, which can be written as

2 $$\mathrm{CO}+H_{2}O\to C0_{2}+H_{2}$$

The methanol synthesis process is conducted at 400–500 °C in the presence of a catalyst. The methanol formation reaction can be represented by

3 $$\mathrm{CO}+2H_{2}↔{\mathrm{CH}}_{3}\mathrm{OH}$$

This crude methanol is purified to 99.9% methanol in a train of distillation columns. This is a very energy-intensive process and produces a large amount of carbon dioxide, typically 0.5 tons of carbon dioxide for every ton of methanol produced.^6^ A portion of this carbon dioxide is produced during the process of making methanol and is vented, while the rest is produced via combustion of hydrocarbons for utility heat.

Figure 2 shows an alternative scheme to produce methanol via dry reforming.^9^ In this scheme, methane reacts with carbon dioxide to produce syngas, which is further converted to methanol. The dry reforming reaction can be represented by the following equation

4 $$CH_{4}+CO_{2}↔2\mathrm{CO}+2H_{2}$$

**Figure 2:**
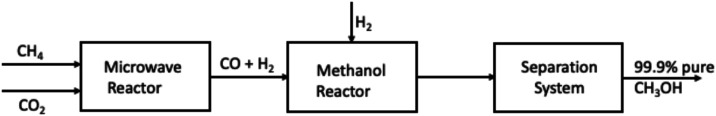
MW dry reforming process to produce methanol.

This process uses carbon dioxide instead of producing it. This carbon dioxide can be obtained via capture from flue gas or directly from the air. Alternatively, a pure stream of carbon dioxide can be generated from a chemical reaction or obtained as a byproduct from another part of the process plant. Carbon dioxide capture technologies have evolved from energy-intensive chemical absorption methods to novel solid sorbent-based processes characterized by low regeneration energy requirements and scalability.^10^ However, carbon dioxide capture is an energy-intensive step even when it is recovered from concentrated sources of carbon dioxide, requiring as much as 100 MW of energy using typical capture processes based on alkanolamines from the flue gases of a 500 MW coal fired power plant.^11^ Dwivedi et al. evaluated a novel trireforming process using flue gas and methane to produce synthesis gas, which is then converted to methanol and showed a significant improvement in gross margin and net percentage of carbon dioxide valorized when the flue gas is utilized directly without first separating carbon dioxide.^12^ Furthermore, they considered the benefits of combining oxy-fuel combustion with the trireforming coupled methanol production process and showed a substantial improvement in both carbon dioxide valorization potential as well as profitability.^13,14^

The dry methane reforming (DMR) reaction is thermodynamically favorable at high temperatures (800–1000 °C) and low pressures and requires a suitable catalyst to overcome the kinetic limitations and avoid the formation of carbon deposits. Thermally driven DMR is carried out in multitubular reactors, where the tubes are heated by an external furnace. Thermal DMR has not been commercially implemented on an industrial scale due to its high operating costs stemming from intensive energy requirements and short catalyst lifetime.^15^

An alternative to conventional tubular reactors is the use of plasma reactors. In plasma reactors, electron-driven reactions induced by plasma enable the activation of chemically inert molecules under relatively mild conditions. Recently, significant progress has been made in understanding plasma-induced reactions, plasma-catalyst interactions, and reactor development to enhance energy efficiency and conversion when applied to carbon capture and utilization.^16,17^ Plasma not only enhances carbon dioxide conversion but also induces desorption from sorbents and assists in the activation of membranes. Furthermore, the conversion can be enhanced through tailor-designed operation schemes, which are unique features of these novel plasma systems. However, research in this field is still in the proof-of-concept stage, making it premature to analyze overall process economics and sustainability.^16^

Another alternative to plasma reactors is the use of microwave reactors, which have the same advantage as plasma reactors in terms of reduced energy usage. Operating on the principles of electromagnetic radiation in the microwave frequency range, these reactors have garnered significant attention due to their ability to induce rapid and selective heating of reaction mixtures.^5^ At the core of microwave reactors lies the interaction between microwave radiation and polar molecules within a reaction mixture. Microwaves, a form of nonionizing electromagnetic radiation, possess the unique property of being absorbed by molecules that exhibit a permanent dipole moment. This absorption results in the generation of molecular rotational energy, thereby inducing rapid and uniform heating throughout the reaction medium. The key to microwave reactors’ efficacy lies in the concept of volumetric heating. Unlike traditional heating methods, such as convection or radiation, where heat is transferred from the external surface of the reaction vessel toward the interior, microwave reactors heat the entire volume of the reaction mixture simultaneously. This volumetric heating leads to a rapid temperature elevation, enabling swift initiation of chemical reactions. The selective heating capacity of microwave reactors is rooted in the preferential absorption of microwave energy by polar reactants or reaction intermediates. Substances with higher dielectric constants tend to absorb microwave radiation more effectively. Consequently, reactions involving polar or ionic compounds, as well as those characterized by significant dipole moments, are often accelerated under microwave irradiation. Microwave reactors operate within a controlled and highly customizable environment using electrical energy, which can be obtained from renewable resources in the future. Reaction conditions, including temperature, pressure, and reaction time, can be precisely regulated to optimize reaction kinetics. Additionally, microwave reactors are often equipped with various safety mechanisms to prevent overpressurization and ensure operator well-being.^18^ This provides the motivation to consider the use of microwave reactors for dry reforming of carbon dioxide to produce syngas and utilize this syngas to produce methanol.

The dry reforming process requires additional hydrogen for conversion to methanol. The most common industrial process for producing hydrogen involves steam methane reforming, which is also an energy-intensive process that generates carbon dioxide.^18^ Thus, switching to dry reforming may not necessarily lead to savings in energy or the reduction in carbon dioxide emissions, and it is necessary to account for these additional requirements in process calculations.

Figure 3 shows a scheme that combines chemical looping with dry reforming. A chemical looping scheme is utilized in which a feed of methane and steam is converted into a pure stream of hydrogen and a pure stream of carbon dioxide.^19^ In this 3-reactor setup, methane is used as a reducing agent for the metal oxide to abstract lattice oxygen. Hydrogen is generated from the steam water splitting reaction. The carbon dioxide produced is free of nitrogen, and this method essentially achieves clean hydrogen production by separating the oxidation steps from the fuel reaction in a cyclic process. Furthermore, water generated in the chemical looping step is condensed and recycled. Integration of this scheme with dry reforming has the potential to reduce the emissions of carbon dioxide and save energy. The hydrogen is sent to the methanol reactor, and carbon dioxide is sent to the dry reforming microwave reactor. This integrated system has the potential to reduce the emission of greenhouse gas carbon dioxide while utilizing less energy in the dry reforming process.

**Figure 3:**
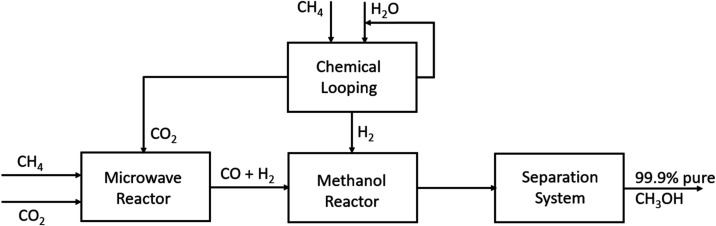
Chemical looping integrated with dry reforming.

## 3 Methodological Framework and Integrated Process Modeling

In a previous publication, a detailed model for Natgasoline’s conventional process (shown in Figure 1) to make methanol was developed in the ASPEN Plus simulation environment and validated with plant data.^8^ This conventional process converts methane into methanol with a production rate of 14,201 lbmol/h of 99.9% pure methanol. For the purposes of this study, this production rate is used as the basis for all process calculations. The feed to the conventional process consists of 14,938 lbmol/h of natural gas, and specific details on feed conditions, process temperatures, pressures, and flow rates were derived from operational data provided by Natgasoline LLC. In the conventional process, methane reacts with steam within a multitubular reactor at 975 °C and 36 bar, producing synthesis gas (syngas) and carbon dioxide. This syngas stream is subsequently directed to a methanol reactor, which operates at 254 °C and 76 bar. The resulting crude methanol undergoes further purification through a series of distillation columns to achieve the desired purity level of 99.9% (by mol) of a production rate of 14,201 lbmol/h of methanol.

The same modeling methodology is utilized to develop a simulation model for the novel microwave-assisted dry reforming process.^8^ The chemical looping process is modeled as a three-reactor system in equilibrium and is integrated with the dry reforming process.^19^ The modeling effort is divided into the following sections: (1) steady-state model development, (2) heat exchanger network (HEN) design, and (3) economic evaluation. An overall integrated process is developed in the ASPEN Plus environment for the steady-state simulation by combining process information, feed specifications, and operating conditions from the literature.^20^ Next, a detailed heat exchanger network (HEN) is developed via ASPEN Energy Analyzer (AEA) v11.0 software. A HEN grid is obtained from heat integration analysis that meets the targeted utility consumption, capital investment, and surface area requirements. Finally, the profitability of the integrated process is determined by performing an economic analysis. The most widely used economic indicator to assess the economic profitability of the production process is the net present value (NPV). NPV is a financial metric that calculates the present value of all future cash flows from a project, taking into account both the initial capital cost (CAPEX) and ongoing operating costs (OPEX) by discounting them to their current worth using a chosen discount rate; essentially, it shows the net profit an investment is expected to generate today, considering the time value of money, where a positive NPV indicates a profitable project and a negative NPV signifies a loss-making one.^21^ The major technical details of the integrated process, the HEN model, and the economic analysis are described in detail in the succeeding sections.

## 4 Integrated Process Modeling

Figure 4 shows a detailed process flow diagram that is developed from the block flow diagram shown in Figure 3. In Figure 4, subsystem 1 represents the dry reforming and methanol formation process, while subsystem 2 represents the chemical looping process.

**Figure 4:**
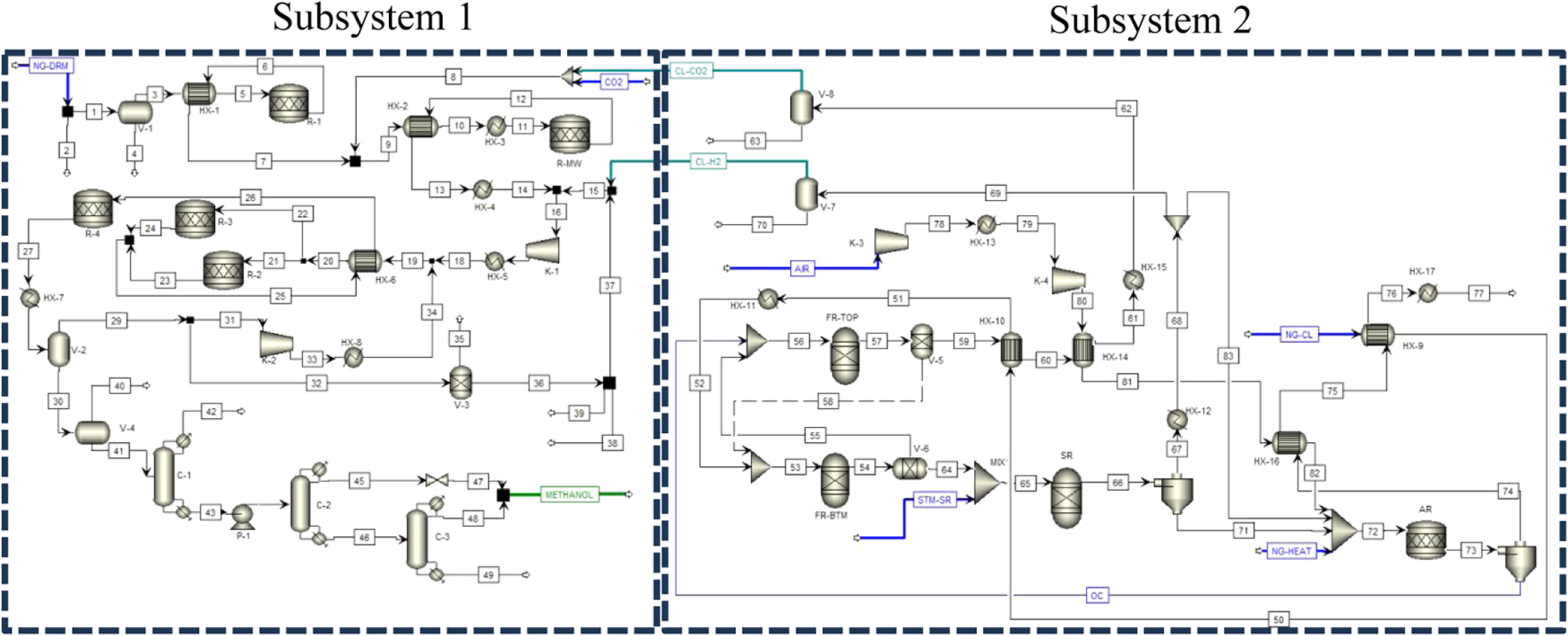
Process flow diagram of integrated dry reforming and chemical looping processes.

In Subsystem 1, the methanol production process utilized in this research is modeled similarly to the commercial setup at Natgasoline LLC, with adjustments to the syngas production, feed conditions, and reactor configurations. The syngas feed for methanol synthesis is through a microwave-assisted dry reforming reactor, where methane (CH_4_) and recycled carbon dioxide (CO_2_) are converted to a syngas stream. This process is conducted at 800 °C and 1 bar. The H_2_ ratio in the syngas is optimized by supplementing it with hydrogen generated from the chemical looping process in Subsystem 1. The operating specifications for microwave-assisted syngas production are provided in Table 1.^9^

**Table 1:** Feed Specifications for Syngas and Chemical Looping Processes

	natural gas			water			
stream	NG-DRM	NG-CL	NG-HEAT	STM-SR	CO_2_	OC	AIR
temperature (°C)	30	25	25	230	30	970	25
pressure (bar)	1	2	1	2	1	2	1
flow rate [kmol/h]	2648	3320	100	8597	1168	71,297	133,446
Composition (mol %)							
methane	94.7						
ethane	3.84						
propane	0.82						
butane	0.15						
CO_2_	0.25				100		
nitrogen	0.24						78.99
O_2_							21.01
H_2_O				100			
OC (CaFe_2_O_4_)						100	
Total	100			100	100	100	100

The conditioned syngas is then compressed to approximately 76 bar and distributed across two parallel methanol synthesis reactors, R-2 and R-3, each operating at 254 °C and 76 bar. Here, carbon monoxide and carbon dioxide undergo hydrogenation in the presence of a Cu/Zn-based catalyst to produce methanol and water. The product gas from reactors is then preheated by exchanging heat with the reactor feed stream before undergoing a final conversion step in Reactor R-4, operated at 220 °C and 71 bar. This additional reactor stage helps maximize the methanol yield by enhancing the conversion of residual carbon monoxide and carbon dioxide. The reactor outlet gas from R-4, containing methanol, water, and unreacted gases (H_2_, CO, CO_2_, CH_4_, and inert gases like N_2_), is directed to Separator V-2, where crude methanol is separated at 40 °C and 67 bar. A portion of the unreacted gases is recycled back to the reactors (R-2 and R-3) to improve the process efficiency, while a portion is purged. The purification of crude methanol is achieved through a series of steps involving Separator V-4 and three distillation columns (C-1, C-2, and C-3): Table 2 summarizes the column specifications for methanol purification. In Separator V-4, crude methanol is depressurized, allowing off-gas, which mainly consists of carbon dioxide, hydrogen, and methane, to be vented. The liquid phase is directed to Column C-1 for initial purification. Column C-1 separates residual carbon dioxide and trace gases, which are flared, while the methanol-rich bottom stream is pumped into Column C-2 for further separation. In Column C-2, methanol of approximately 99.99% purity is collected as the distillate, while the remaining mixture of methanol and water proceeds to Column C-3, where the purification is complete by separating methanol and water, producing a final methanol product with a purity of 99.99 mol %.

**Table 2:** Specifications for Methanol Purification Columns

	C-1	C-2	C-3
purpose	distillation	distillation	distillation
No. of stages	12	47	36
feed stage	8	39	26
feed temperature (°C)	12	87	135
pressure (bar)	2	8	1
reflux ratio	1.82	1.27	0.74
condenser duty (MJ/h)	–14,404	–223,078	–224,991
condenser temperature (°C)	13	127	69
distillate rate (kmol/h)	174.64	3857.41	3410.61
reboiler duty (MJ/h)	86,194	269,039	189,151
reboiler temperature (°C)	86	135	107
bottom rate (kmol/h)	8161.94	8016.45	4405.4

In Subsystem 2, the chemical looping process is configured as a three-reactor, countercurrent setup to enable hydrogen production and carbon dioxide capture, based on the conditions outlined in the work of Riley et al.^19^ The looping system uses CaFe_2_O_4_ as an oxygen carrier, which alternates between oxidized and reduced forms to facilitate methane and steam conversion in a controlled series of reduction and oxidation cycles. Process feed conditions and specifications for this setup are summarized in Table 1. Conditions such as temperature, pressure, and gas flow rates are adapted from Riley et al.^19^ but are further adjusted to meet the specific H_2_ demand of our methanol synthesis setup. The chemical looping system operates as follows: In the fuel reactor (FR), natural gas is introduced to react with oxidized CaFe_2_O_4_, producing carbon dioxide and water while reducing the oxygen carrier. This stage is conducted at elevated temperatures, allowing efficient conversion of methane into carbon dioxide and water that can be further processed. In the air reactor (AR), the reduced CaFe_2_O_4_ is reoxidized by reacting with air, regenerating the oxygen carrier. In the steam reactor (SR), the reoxidized CaFe_2_O_4_ interacts with steam, producing hydrogen with some unconverted steam. The operating conditions for each reactor in the chemical looping process are listed in Table 3. The hydrogen generated in this stage is directed to Subsystem 1 to supplement the syngas stream, achieving the necessary hydrogen ratio for methanol synthesis.

**Table 3:** Reactor Operating Conditions in Chemical Looping

reactor	subplant	function	temperature (°C)	pressure (bar)
fuel reactor (TOP-FR)	1	reduce oxygen carrier	960	2
fuel reactor (BTM-FR)	1		870	2
air reactor (AR)	1	oxygen carrier regeneration	970	2
steam reactor (SR)	1	H2 production	875	2

## 5 Process Simulation

### 5.1 Process Simulation of Methanol Synthesis

The methanol synthesis process was simulated using Aspen Plus, v11,^20^ utilizing process parameters sourced from the literature. Two thermodynamic models were employed to ensure an accurate representation of process behavior: RK-SOAVE was used for the syngas generation section, while NRTL was applied to the methanol synthesis stage. The process starts with feed preparation in the hydrodesulfurization unit, where natural gas is treated to remove H_2_S, organic sulfur, and odorizing agents. This step prevents sulfur poisoning of catalysts used in the reforming and methanol synthesis stages. The hydrodesulfurization reactions were simulated in a stoichiometric reactor (R-1). Since the feed gas contained no sulfur compounds, the reactor effluent composition remained unchanged from that of the feed. The reactor effluent was subsequently used to preheat the reactor feed stream before being mixed with a carbon dioxide stream. The resulting mixture was heated to 610 °C at 1 bar using energy recovered from the microwave reactor effluent and then to 800 °C using energy supplemented by a heater. The preheated mixture entered the microwave-assisted dry reforming reactor, which is modeled as a stoichiometric reactor that operates at 800 °C and 1 bar. In this reactor, methane underwent reforming reactions, converting it into syngas composed of 40.3% H_2_, 41.5% CO, 17% CO_2_, and 0.57% CH_4_, achieving a methane conversion rate of 97%.^22^ To achieve the stoichiometric conditions required for methanol synthesis, the syngas stream was supplemented with an external hydrogen source, which provides sufficient hydrogen to result in a stoichiometric number (SN) of 2 for the methanol reactor. The conditioned syngas was compressed to 76 bar and preheated to 254 °C before being distributed to two twin parallel methanol synthesis reactors (R-2 and R-3). The effluent streams from R-3 and R-4 were combined and passed through a reactor (R-4) for a final conversion. The combined reactor outlet was then directed to Separator V-2, where crude methanol was separated from unreacted gases and trace impurities. A portion of the unreacted gases (split ratio: 26.5%) was purged to control inert gas accumulation, while the remaining portion was pressurized and recycled to R-2 and R-3. The crude methanol stream, exiting the separator at a flow rate of 8,386.84 kmol/h, 67 bar pressure, and 40 °C, contained 77.3 mol % methanol. This stream was sent to a series of distillation columns (C-1, C-2, and C-3) for purification. The columns, modeled using RadFrac, contained 12, 47, and 36 stages, respectively, operating at pressures of 2, 8, and 1 bar. The optimal reflux ratios were determined to be 1.82, 1.27, and 0.74, respectively, for the three columns. A partial vapor condenser was used for C-1 to vent the off-gas, while total condensers were employed for C-2 and C-3 to ensure methanol recovery as a liquid product. Column designs were optimized using mole recovery specifications by varying the reflux ratio and distillate-to-feed (D/F) ratio. Figures 5, 6, and 7 show the temperature and composition profiles in columns C-1, C-2, and C-3.

**Figure 5:**
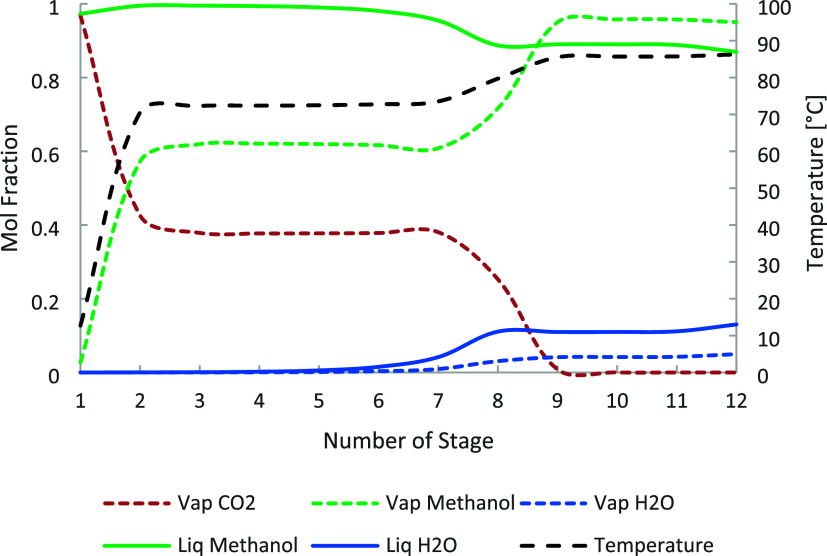
C-1 temperature and composition profile for methanol, water, and carbon dioxide.

**Figure 6:**
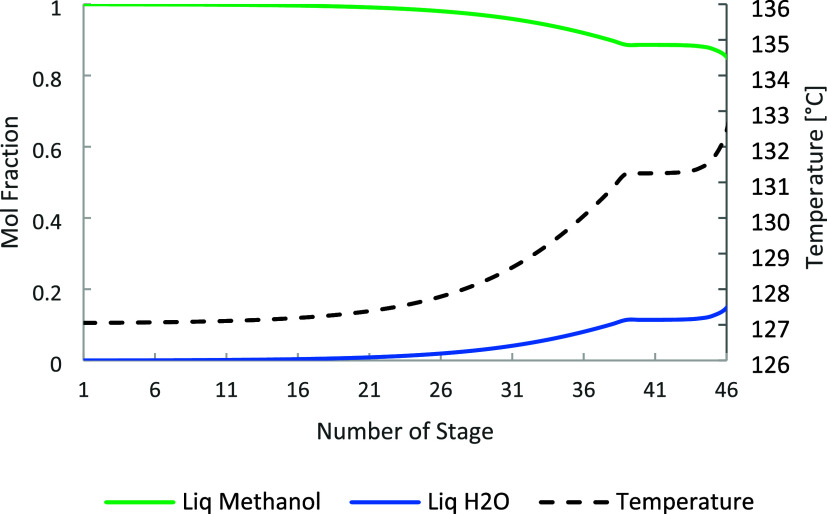
C-2 temperature and composition profile for methanol and water.

**Figure 7:**
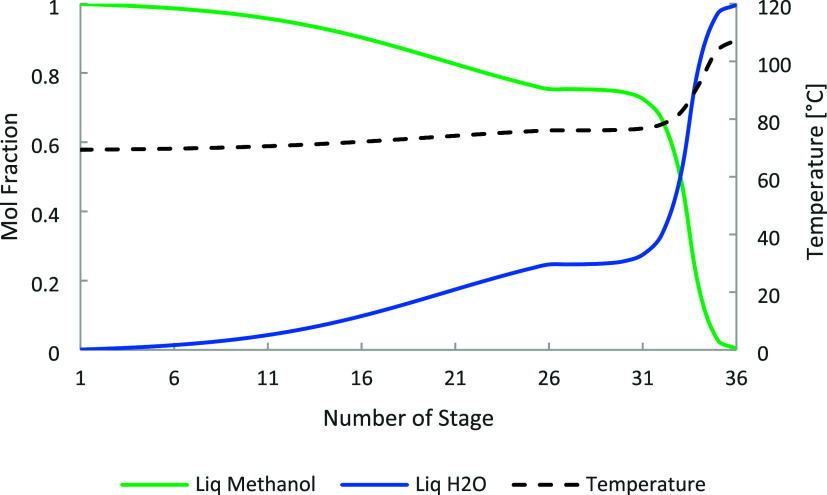
C-3 temperature and composition profile for methanol and water.

### 5.2 Process Simulation of Chemical Looping

The three-reactor configuration was simulated in Aspen Plus, version 11, using the Peng–Robinson thermodynamic package for gas-phase components. The thermodynamic properties of the solid phases were imported separately from the literature. This configuration includes a fuel reactor (FR), steam reactor (SR), and air reactor (AR), operating in a cyclic chemical looping process with CaFe_2_O_4_ as the oxygen carrier. The feed rates were adjusted to meet the hydrogen production requirements for the dry reforming of the methanol process. The FR operates as the first step in the cycle, where 7,320 lbmol/h of natural gas reacts with oxidized CaFe_2_O_4_. This process reduces the carrier while producing a stream composed primarily of CO_2_ and water. The FR consists of two zones, FR-TOP and FR-BTM. The FR-TOP receives the oxidized carrier from the AR and the gas effluent from FR-BTM, operating at 960 °C and 2 bar. The FR-BTM receives natural gas and partially reduced solids from FR-TOP, operating at 830 °C and 2 atm. Both zones were modeled as adiabatic RGibbs reactors to simulate equilibrium reactions. The gas stream exiting FR-TOP undergoes condensation for steam removal, resulting in a CO_2_-rich stream with a high purity. This unit, along with multicompressor stages, ensures a high-purity carbon dioxide product stream of 7,163 lbmol/h, which is approximately 73% of the carbon dioxide required for the methanol dry reforming process. The reduced carrier exiting FR-BTM is sent to the SR, where water splitting occurs. In the SR, steam reacts with reduced CaFe_2_O_4_ to produce high-purity hydrogen and partially oxidized carrier materials, including Ca_2_Fe_2_O_5_ and Fe_3_O_4_. The SR operates at 875 °C and 2 bar and was modeled as an RGibbs reactor. The resulting hydrogen stream is purified in a condenser, where water is effectively removed, to achieve a product purity of 99.9%. The hydrogen production rate is 22,296 lbmol/h, and when this is combined with the syngas stream that goes to the methanol reactor, a stoichiometric number (SN) of 2 is achieved, thereby meeting the amount required for the methanol dry reforming process. The reduced carrier exiting the SR is mixed with air and natural gas for heating before entering the AR. The air stream, compressed to 3 bar and heated to 861 °C, is combined with the reduced carrier and natural gas (at 25 °C and 1 bar) in a mixer. This mixture is then fed into the AR, where the reduced carrier is regenerated by reacting with air. The AR operates at 970 °C and 2 bar.

### 5.3 Heat Exchange Network

The heat integration network (HEN) for the microwave-assisted dry reforming process with chemical looping was developed to optimize the utility usage and reduce energy costs. The design methodology focused on minimizing the total utility cost from a process systems perspective, utilizing a minimum temperature approach (Δ*T*_m_) of 10.0 °C. Aspen Energy Analyzer (AEA) V11.0 was used for the HEN development; this software utilizes a pinch analysis-based optimization algorithm to compute heat load matches, surface areas, and cost targets. The optimization objective function was formulated as a mixed-integer linear programming (MILP) problem with the aim of minimizing utility costs. The optimization considered multiple matches rather than a single match, employing dummy elements with zero assignments to balance the number of hot and cold streams when necessary. This ensured a square matrix that was suitable for MILP solution generation. Energy-saving opportunities were analyzed separately for each subsystem and for the overall integrated process. Results were compared to the base case scenario with no heat integration.

The integrated process was divided into two subsystems for the development of the HEN, Subsystems 1 and Subsystem 2. Heat integration was applied within each subsystem independently without sharing energy between subsystems. The heat integration analysis for Subsystem 1 focuses on optimizing thermal energy utilization within the methanol production process by recovering heat from high-temperature process streams and redistributing it to preheat incoming feeds. The high-temperature hot streams (above 600 °C) primarily originate from reactor effluent gases and heat exchangers associated with syngas production. These streams transfer energy to preheated feed gases before they enter key reaction zones, reducing the demand for external heating utilities. Midtemperature heat recovery (200–500 °C) occurs in streams exiting intermediate processing units, contributing to the efficient distribution of heat within the system. At lower temperatures (below 200 °C), residual process heat is utilized for methanol purification and separation, further enhancing the energy efficiency. The heat integration network for Subsystem 2 focuses on optimizing the thermal efficiency by strategically recovering and reusing heat within the process. The analysis evaluates the interactions between hot and cold streams, ensuring that high-temperature effluent gases from the chemical looping system effectively preheated incoming reactants, minimizing the need for external utilities. The results highlight key energy-saving strategies, including the recovery of high-temperature exhaust heat (above 800 °C) to preheat feed streams as well as mid- and low-temperature heat exchanges that contribute to overall process efficiency.

The overall performance of HEN is summarized in Table 4. The base case simulation revealed a highly energy-intensive process, with a hot utility requirement of 2923 MMBTU/h and a cold utility demand of 3,968 MMBTU/h. The chemical looping subsystem contributed significantly to the cold utility demand due to its high-temperature cycle, which necessitated substantial cooling. The optimized HEN design demonstrated significant reductions in utility requirements. For the integrated subsystems, the hot utility demand decreased to 478 MMBTU/h, representing an 83.6% reduction, while the cold utility demand dropped to 1,482 MMBTU/h, achieving a 62.7% reduction. These results highlight the effectiveness of the HEN methodology in improving energy efficiency.

**Table 4:** HEN Performance

utility requirements [MMBtu/H]	base case	heat integrated	% reduction
hot utility	2923	478	83.60
cold utility	3968	1482	62.70

## 6 Economic Analysis of the Process

The economic analysis focuses on two main factors: (1) capital expenditure (CAPEX) and (2) operational expenditure (OPEX).^21^ CAPEX includes the costs associated with purchasing and installing equipment, piping, plant erection, and civil infrastructure. OPEX includes the costs of raw materials, maintenance, and utilities necessary for plant operation. Capital costs for standard equipment, such as pumps, multistage compressors, vessels, heat exchangers, and distillation columns, were estimated by mapping equipment items from the Aspen Plus plantwide model to the Aspen Process Economic Analyzer (APEA) V11. Costs for the microwave-assisted reactor (MW reactor) were estimated separately due to its novel design. The MW reactor cost was divided into two components: the magnetron (including electrical ancillaries) and the pressure vessel. The magnetron cost was determined based on the power (kW) rating, as shown in Ogunniyan et al.,^23^ and included components such as the cavity, generator, controls, transformers, switches, installation, and setup. The pressure vessel cost was calculated using correlations from Turton et al.,^21^ incorporating material of construction and operating pressure. Additional costs, such as assembly, commissioning, and markup, were escalated to account for the novel status of the MW-assisted reactor technology. Utility requirements, including electricity, cooling water, steam, and raw material flow rates, were obtained from the Aspen Plus process model. Specific utility costs are listed in Table 5, and key economic parameters such as plant life, startup period, and contingency are summarized in Table 6.

**Table 5:** Utilities, Raw Materials, and Products

category	description	value	units
raw material	natural gas	1.8	$/MMBtu
	CO_2_	38.59	$/MT
utility	cooling water	0.378	$/Gj
	Hp steam	5.66	$/Gj
	Lp steam	4.55	$/Gj
	electricity	8.72	c/kWh
products	methanol	785	$/MT
	hydrogen	0.83	$/kg

**Table 6:** Key Economic Parameters

investment parameters	value
contingency	26%
tax rate	40%
desired internal rate of return	10% per year
products and raw material escalation	1% per year
project capital escalation	1% per year
utility escalation	1% per year
economic life of project	10 years
operating hours per year	8000 h
salvage value	20%
O&M expenses	3% per year
G&A expenses	8% per year
plant overhead	50%
working capital percentage	12% per year
startup period	30 weeks
plant capacity	14,201 lbmol/h

Based on the relatively straightforward commissioning process for MW-assisted reactors, a startup period of 30 weeks was estimated. Due to the novelty of MW-assisted reactors and their untested performance at large scale, a 26% contingency factor was applied. A 10-year plant life was assumed, reflecting the emerging nature of the MW-assisted reactor technology.

The economic feasibility of the flowsheets was assessed using the net present value (NPV). A positive NPV indicates that the project is economically viable. The study compares conventional methane steam reforming (SMR) to different cases of microwave-assisted dry reforming of methane (MW-DRM). The cases considered are as follows: Case 0: Conventional SMR (Natgasoline), Case I: MW-DRM with chemical looping for hydrogen production (this requires additional carbon dioxide), Case II: MW-DRM with chemical looping for carbon dioxide production (this produces excess hydrogen). The comparison highlights the economic performance of MW-DRM configurations against that of traditional SMR, emphasizing the potential of MW-assisted technology integrated with chemical looping for both hydrogen and carbon dioxide production.

### 6.1 Case 0: Conventional Steam Methane Reforming (Natgasoline)

The economic analysis of the conventional steam methane reforming (SMR) process, based on the Natgasoline model, provides a baseline for comparison.^8^ The total project capital cost is $234.44 MM, and the total operating cost is $356.71 MM/year, which includes a raw material cost of $177.78 MM/year and a utility cost of $178.93 MM/year, with a methane utilization rate of 1,023,739 tons/year and a carbon dioxide emission rate of 202,364 tons/year. The net present value (NPV) for this case is $2,760 MM, and the levelized cost of methanol production is $**248/ton**, which is consistent with the value reported in the literature.^6^

#### 6.1.1 Case I: MW-DRM with Chemical Looping for Hydrogen Production

The economic analysis of the proposed methanol production process evaluates Subsystems 1 and 2. Subsystem 1, responsible for methanol production, has a total capital cost of $382.01 MM and annual operating costs of $307.376 MM, driven primarily by raw material costs of $106.32 MM/year and utility costs of $178.94 MM/year. The NPV for Subsystem 1 is $2,571 MM. The breakdown of CAPEX and the breakdown of OPEX are shown in Table 7.

**Table 7:** MW-DRM Cost Breakdown

CAPEX		OPEX	
unit		parameter	
MW reactor	210.04 $MM/yr	raw material	106.32 $MM/yr
EPC, instruments, contingency, others	171.98 $MM/yr	utility	178.94 $MM/yr
		O&M, G&A	22.12 $MM/yr

Subsystem 2, responsible for hydrogen production and partial CO_2_ capture using chemical looping, has a total capital cost of $133.66 MM, with annual operating costs of $178.75 MM/year, including raw material costs of $55.93 MM/year and utility costs of $100.20 MM/year. The net present value (NPV) for Subsystem 2 is −$1,132 MM, reflecting the system’s focus on producing intermediates (H_2_ and CO_2_) rather than generating direct revenue. Cost of CAPEX and OPEX are listed in Table 8.

**Table 8:** Chemical Looping Cost Breakdown

CAPEX		OPEX	
unit		parameter	
reactors	51.61 $MM/yr	raw material	55.93 $MM/yr
compressors	31.93 $MM/yr	Utility	100.20 $MM/yr
heat exchangers	15.76 $MM/yr	O&M, G&A	10.21 $MM/yr
EPC, contingency, others	34.36 $MM/yr		

When combined, the integrated process achieves a total capital cost of $515.68 MM, annual operating costs of $486.13 MM/year, and a combined NPV of $1,439 MM and levelized cost of methanol is **$382/ton**. The total methane utilization rate for the chemical looping as well as the dry reforming is 788,439 tons/year, which is substantially less than the methane utilization in the conventional process (1,023,739 tons/year). Furthermore, there is a net carbon dioxide consumption rate of 1,543,962 tons/year, and there is no net production of carbon dioxide from the process, whereas the conventional process produces 202,364 tons/year of carbon dioxide.

The economic sensitivity analysis of net present value (NPV) and levelized cost of methanol (LCOM) to variations in the market price of CO_2_ ranges from $–24/ton to $38.6/ton. The results are presented in Figure 8. Negative values of CO_2_ prices are also considered to represent scenarios where CO_2_ producers pay consumers for CO_2_ utilization. It is observed that NPV decreases steadily as the carbon dioxide price increases. At a carbon dioxide cost of $0/MT, the NPV is $1,490 MM, and it declines to $1,439 MM at a carbon dioxide cost of $38.6/MT.

**Figure 8:**
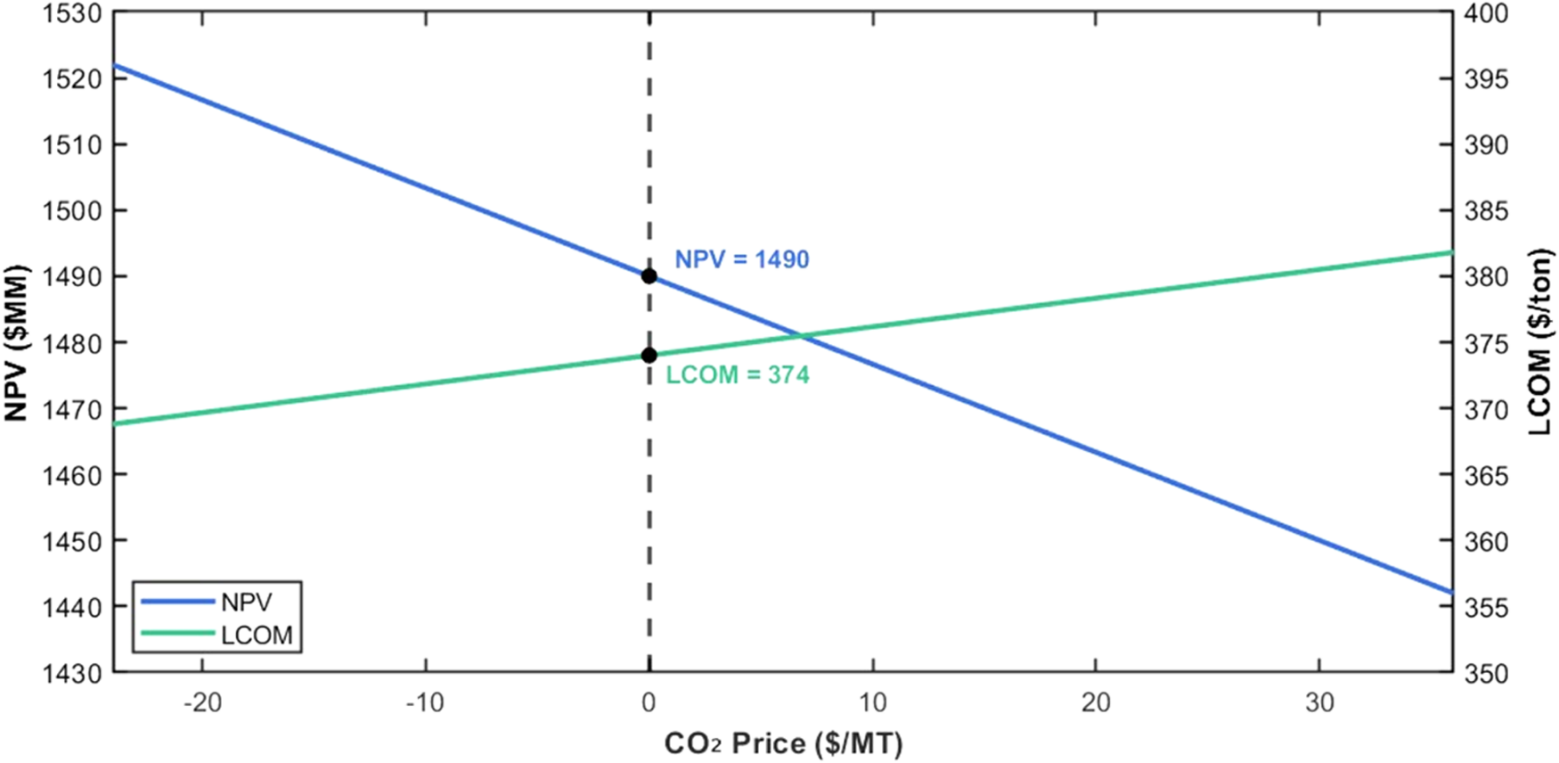
Impact of the carbon dioxide price on NPV and LCOM in case I.

This trend highlights the impact of carbon dioxide cost on project profitability when chemical looping is partially utilized. LCOM increases linearly with increasing carbon dioxide prices. Starting from $374/ton at $0/MT, the LCOM increases to $382/ton at $38.6/ton. The upward trend reflects the increasing contribution of carbon dioxide costs to the total production cost. These results emphasize that while partial chemical looping significantly reduces dependence on purchased carbon dioxide, it does not produce sufficient carbon dioxide for the process, and the cost of the remaining purchased carbon dioxide still affects the overall economic performance of Case I.

The MW reactor, as a novel technology, represents a significant portion of the capital investment in Case I. Due to its developmental stage, the cost of the MW reactor is relatively high compared to that of traditional process equipment. To understand its influence on the economic feasibility of the process, a sensitivity analysis was conducted by varying the MW reactor cost from 100% of the estimated cost to progressively reduced values (75, 50, 25, and 0%). The results are presented in Figure 9.

**Figure 9:**
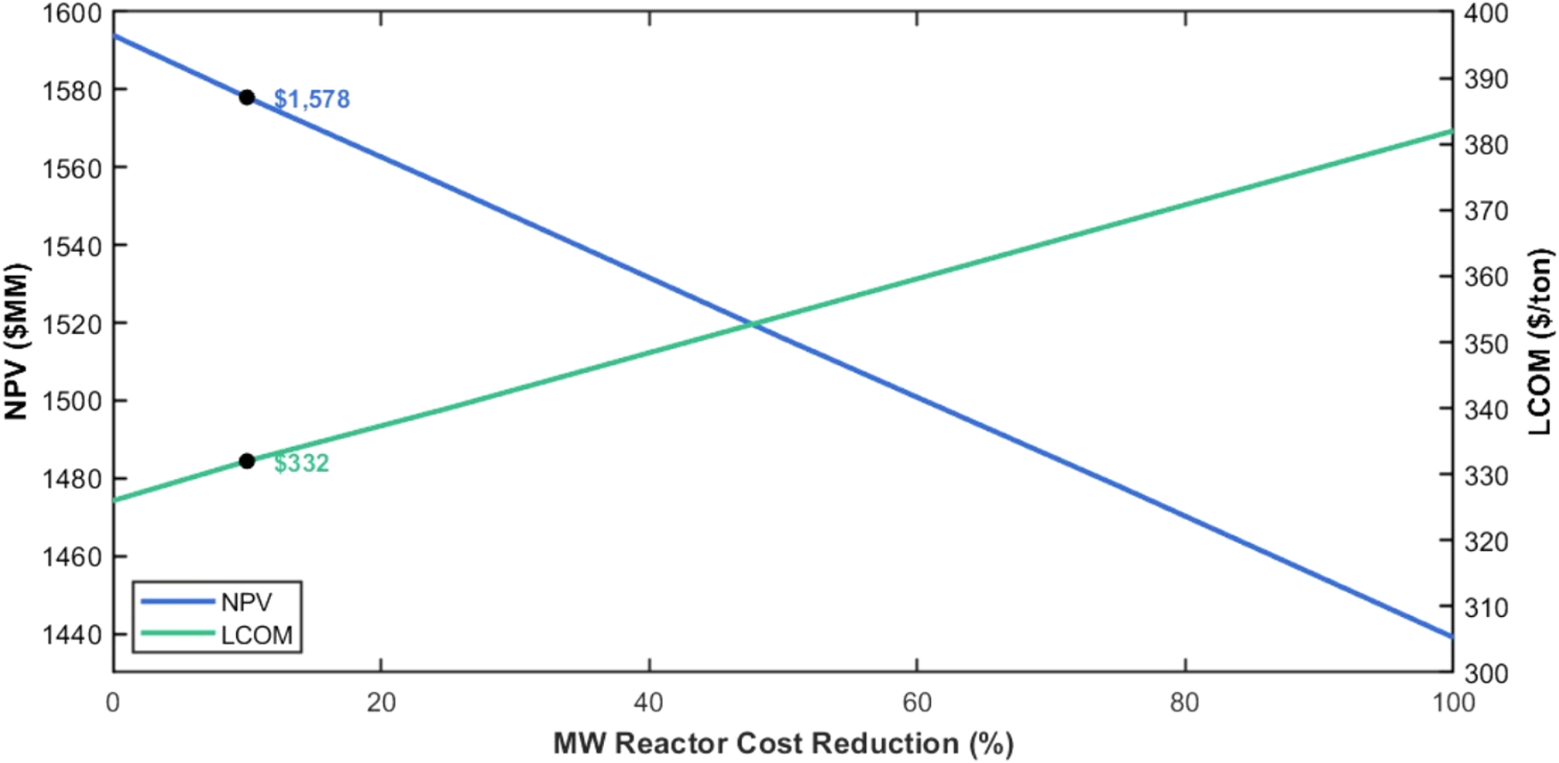
Relationship among the MW reactor cost, NPV, and LCOM.

The analysis shows that as the MW reactor cost decreases, the economic performance of the process improves significantly. At 100% of the MW reactor cost, the NPV is $1,439 MM, and the levelized cost of methanol is $382/ton, reflecting the high capital burden of the reactor. Reducing the reactor cost to 75% increases the NPV to $1,478 MM and decreases the levelized cost of methanol to $368/ton. If the microwave reactor cost were to be estimated to be the same as a conventional fixed bed reactor, the NPV would significantly increase to $1,578 MM, and the levelized cost of methanol would drop to $332/ton. This demonstrates that the high cost of the MW reactor is a key factor limiting the economic feasibility of Case I. Advancements in MW reactor technology, reductions in manufacturing costs, or policy incentives to support innovative clean technologies could significantly improve the economic competitiveness of MW-assisted processes.

The use of microwave reactors that operate on electricity in place of conventional reactors that are heated via hydrocarbon combustion indicates that the cost of electricity can have a significant impact on the leveled cost of methanol. In the analysis done above, we assumed an electricity cost of $0.083/kWh. The U.S. Department of Energy has already achieved its target of electricity cost of $0.06/kWh from solar energy and is currently on track to achieve $0.03/kWh by 2030.^7^ Figure 10 shows the effect of electricity cost, assuming that the cost of the microwave reactor is the same as a conventional reactor and the cost of carbon dioxide is zero.

**Figure 10:**
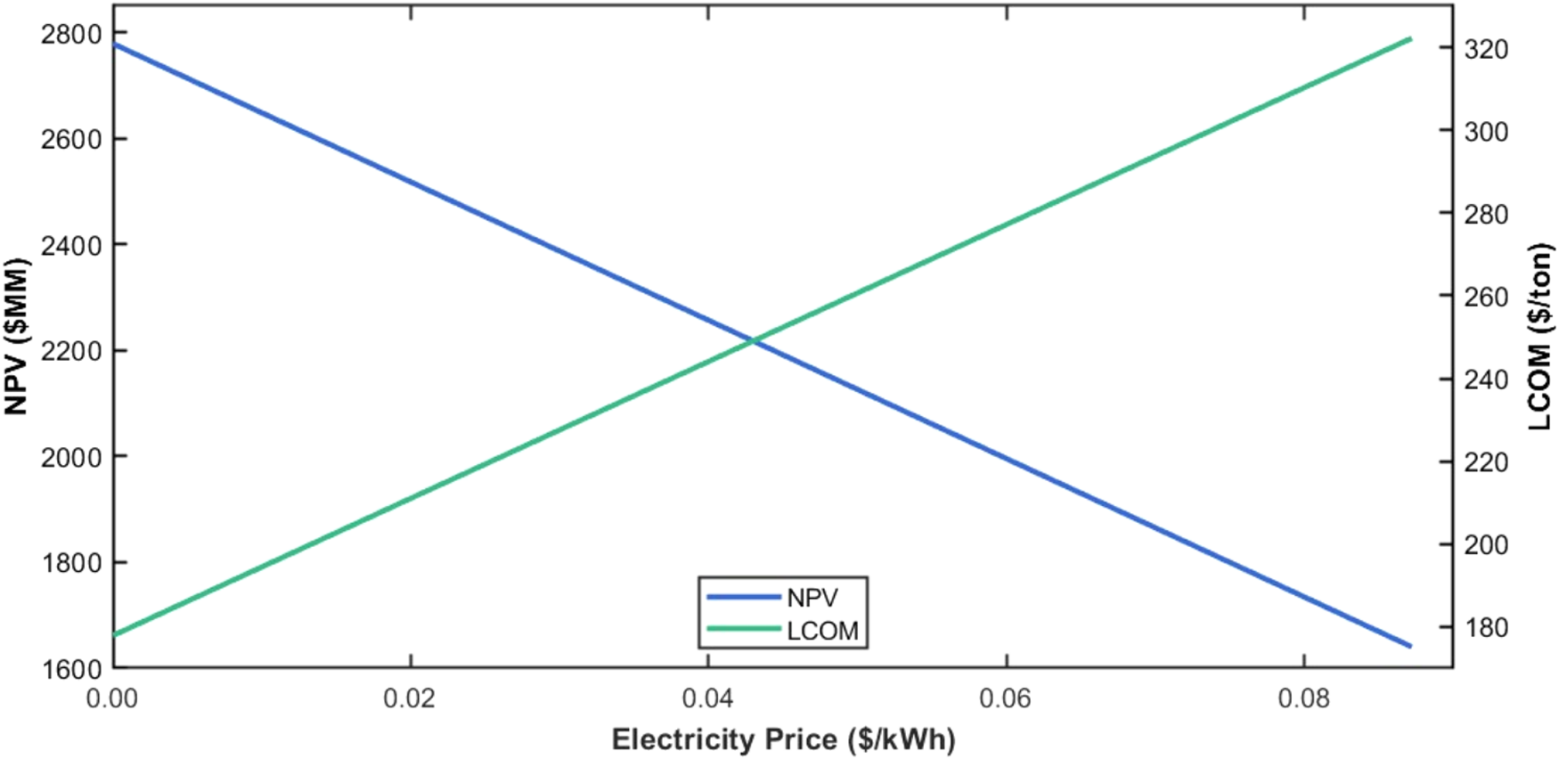
Relationship among electricity cost, NPV, and LCOM.

It is observed that the levelized cost of methanol decreases as the electricity cost decreases. In particular, when the electricity cost is $.03/kWh, the levelized cost of methanol is **$228/ton**, which is lower than the current cost of methanol made from the conventional process.

#### 6.1.2 Case II: MW-DRM with Chemical Looping for Carbon Dioxide Production and Excess Hydrogen

The economic analysis of Case II evaluates Subsystems 1 and 2, where chemical looping is used to produce all of the required carbon dioxide for methanol synthesis, eliminating the need for external purchases. In this configuration, Subsystem 1 was adjusted to supply the necessary carbon dioxide for Subsystem 2, resulting in increased flow rates and associated costs. Additionally, excess hydrogen produced in Subsystem 2 is sold as a byproduct, contributing to the overall economic performance of the integrated process.

Subsystem 1, representing the MW-assisted dry reforming process, has a total capital cost of $382.01 MM and an annual operating cost of $292.85 MM/year. These operating costs include $94.27 MM/year for raw materials and $178.94 MM/year for utilities. The net present value (NPV) for Subsystem 1 is $2661 MM, reflecting the improved economic performance after adjustments to meet the carbon dioxide demands of Subsystem 2. Subsystem 2, focused on hydrogen and carbon dioxide production via chemical looping, has a total capital cost of $209.9 MM and annual operating costs of $205.01 MM/year. Raw material costs amount to $68.26 MM/year, while utility costs contribute $125.03 MM/year. The NPV of Subsystem 2 is $–1,100 MM, primarily due to the high expenses associated with producing the required intermediates and the capital-intensive nature of the process.

The integrated process, combining Subsystems 1 and 2, achieves a total capital cost of $591.92 MM and annual operating costs of $497.87 MM/year. These operating costs include $162.52 MM/year for raw materials and 303.98 MM/year for utilities. The methane utilization rate is 948,628 per year, which is less than the methane utilized in the conventional process. Furthermore, no net carbon dioxide is produced by the process, and the carbon dioxide utilization rate is 1,517,709 tons/year. The integrated system achieves an NPV of $1,561 MM and the levelized cost of methanol at **$434/ton**. The economic feasibility of the combined process is largely supported by the revenue generated from methanol production and the sale of excess hydrogen, which offsets the increased costs in Subsystem 1.

A sensitivity analysis was conducted to evaluate the impact of varying the price of hydrogen on the net present value (NPV) and the levelized cost of methanol (LCOM) of the integrated process in Case II. The results of this analysis are shown in Figure 11.

**Figure 11:**
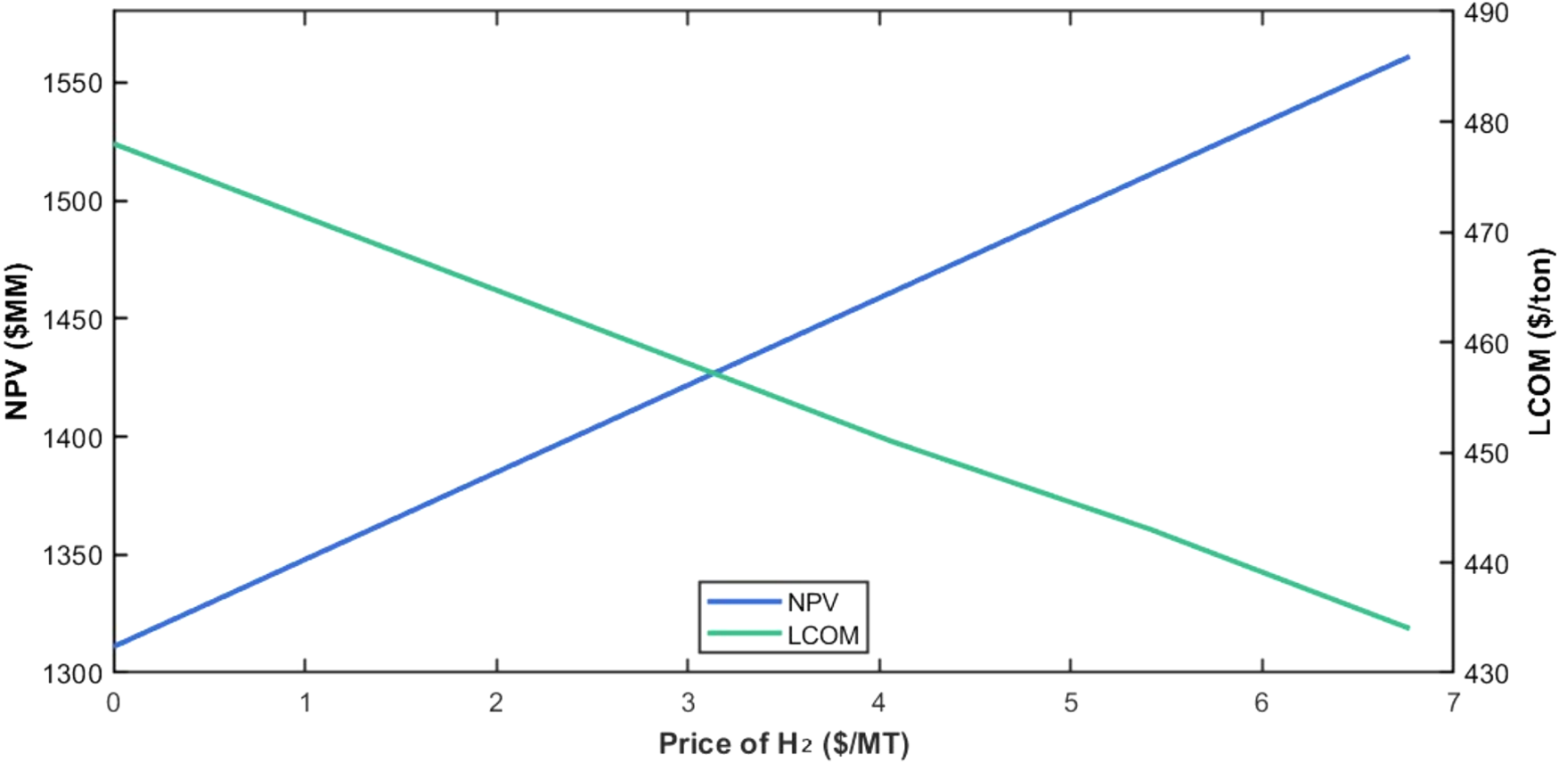
Impact of the Hydrogen Price on NPV and LCOM for case II.

The analysis reveals a positive linear relationship between the selling price of hydrogen and the NPV of the process. At a selling price of $0/MMBtu for H_2_, the NPV is $1,311 MM. As the price of hydrogen increases, the NPV steadily rises, reaching a value of $1,561 MM at $6.77/MMBtu, while the LCOM of methanol decreases from $478/ton to $434/ton. Even though the LCOM of methanol is greater than that in Case I, the NPV is higher than that in Case I because of the extra revenue generated by selling hydrogen. This trend highlights the significant contribution of hydrogen sales revenue to the overall economic performance of the process.

Figures 8–11 clarify how each factor separately influences the feasibility, forming a foundational sensitivity analysis. In practice, operators would optimize multiple parameters simultaneously. However, such multivariable adaptation introduces system-specific complexity that could obscure the direct causal relationships our study emphasizes and will be considered separately in a future publication.

Figure 12 shows that the electricity price emerges as the most impactful variable on both NPV and LCOM, highlighting its critical role in determining the overall economic viability of MW-DRM for methanol systems.

**Figure 12:**
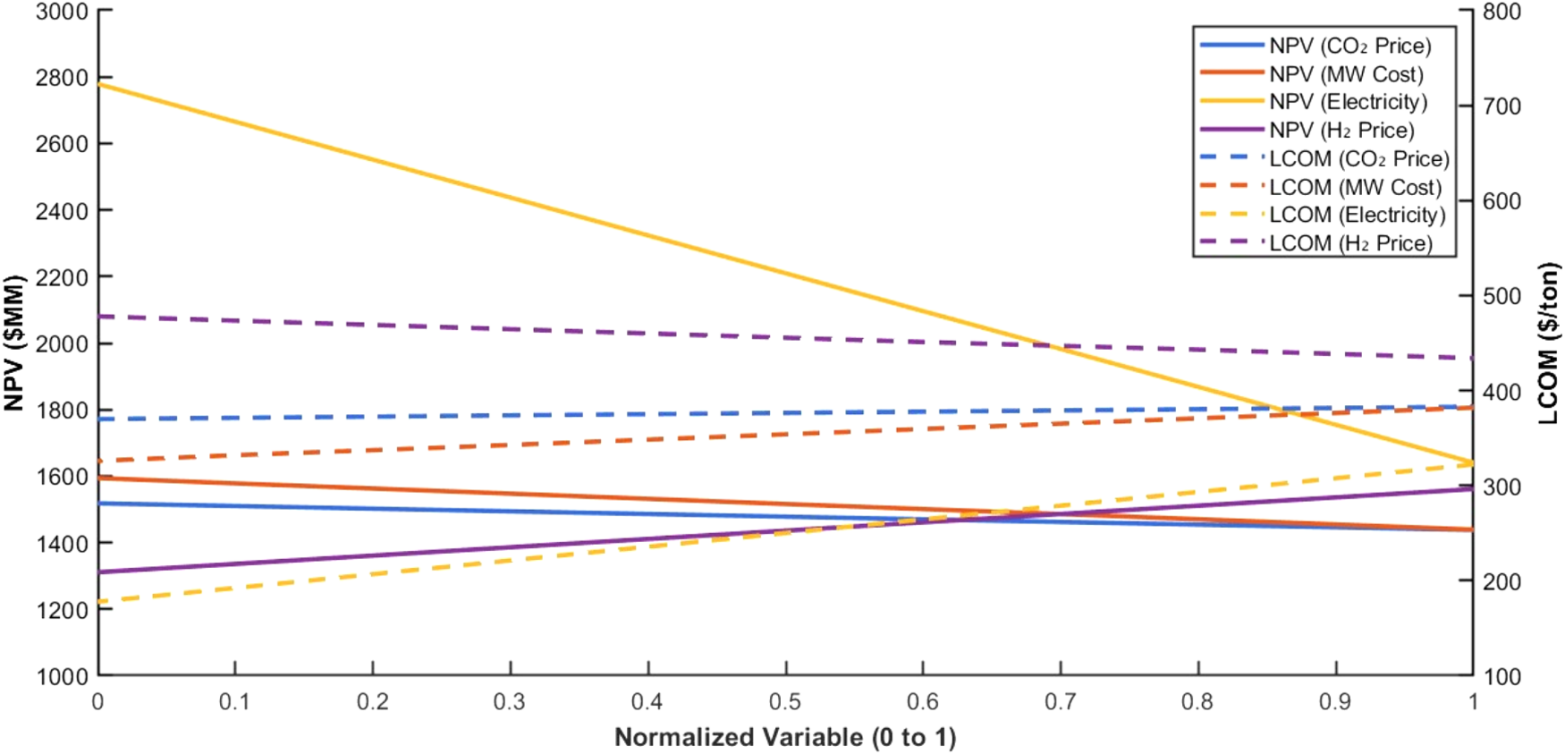
Normalized sensitivities of NPV and LCOM to key input variables.

The comparison also reveals significant differences in the economic performances of the three cases. Case 0, the baseline SMR process, achieves the highest NPV due to its low capital and operating costs. However, it is also associated with high carbon dioxide emissions (202,364 tons/year), making it less favorable in terms of environmental impact. Case I, which integrates chemical looping for hydrogen production, demonstrates a substantial reduction in carbon dioxide emissions and decreases reliance on external H_2_ sources. Furthermore, the cost of methanol varies between $228/ton and $434/ton, which is substantially below the goal of $800/ton set by the United States Department of Energy. Case II, which implements full chemical looping, achieves full independence from external carbon dioxide requirements and generates revenue from excess hydrogen. Despite the higher capital and operating costs, Case II results in a slightly increased NPV compared to Case I due to revenue generated by selling the excess H_2_ and presents significant environmental benefits through reduced carbon dioxide emissions.

Another metric to compare the efficiency of the three processes is to consider the net percentage of carbon dioxide valorized (NPCV), as defined in Dwivedi et al.^12^ NPCV is a measure of carbon dioxide utilized in the process while accounting for emissions through purge streams of unconverted methane, carbon monoxide, and methanol along with carbon dioxide. In the case of partial chemical looping integration (Case I), some carbon dioxide is added from an external source, while some carbon dioxide is produced internally via chemical looping. In full chemical looping with hydrogen sales (Case II), all of the required carbon dioxide is produced internally via chemical looping. The NPCV calculations take both internally generated and externally generated carbon dioxide into account. In the baseline steam methane reforming (Case 0), the NPCV is zero because none of the carbon dioxide produced in the process is utilized toward the production of methanol. The NPCV in partial chemical looping (Case I) is 88.13%, while it is 87.78% for full chemical looping (Case II), which shows the significant valorization potential of dry reforming.

## 7 Conclusions

This study evaluated the economic and environmental performance of three methanol production configurations: baseline steam methane reforming (Case 0), partial chemical looping integration (Case I), and full chemical looping with hydrogen sales (Case II). The analysis revealed trade-offs among economic feasibility, capital investment, operational costs, and carbon dioxide emissions.

Case 0 achieved an NPV of $2760 MM and a levelized cost of methanol (LCOM) at $248/ton due to its relatively low CAPEX and OPEX. However, it resulted in the highest methane utilization rate of 1,023,739 tons per year and the highest carbon dioxide emissions rate of 202,364 tons/year, highlighting its environmental limitations. Case I, integrating chemical looping for hydrogen production, has a methane utilization rate of 788,439 tons/year. It does not produce carbon dioxide but utilizes 1,660,645 tons/year of carbon dioxide. Additional carbon dioxide is required, and a sensitivity analysis revealed that reducing the MW reactor cost to levels comparable to conventional reactors as well as reducing electricity costs could improve the NPV and LCOM significantly, enhancing its economic competitiveness. An economic analysis indicates an LCOM between $228/ton to $382/ton. Case II, which utilized full chemical looping to eliminate external carbon dioxide and generates revenue from hydrogen sales, indicates an NPV of $1,561 MM and an LCOM of $434/ton while utilizing 948,628 tons/year of methane.

Overall, the results demonstrate that transitioning from conventional SMR to MW-assisted dry reforming with chemical looping can provide substantial environmental benefits by utilizing a lower amount of the nonrenewable resource methane while eliminating the production of carbon dioxide from the process. The use of microwave reactors rather than conventionally heated reactors has the potential to further reduce carbon dioxide emissions if renewable resources, such as solar or wind energy, are utilized for electricity generation for powering the microwave reactors. Both Case I and Case II demonstrate the significant potential to utilize carbon dioxide and provide methanol below the goal of $800/ton set by the United States Department of Energy.

The significant methane utilization and carbon dioxide reductions achieved in Cases I and II highlight the potential of MW-assisted dry reforming integrated with chemical looping in enabling cleaner and more sustainable industrial processes. However, the economic viability of these configurations heavily depends on advancements and cost reductions in the MW-assisted reactor technology. Two commonly used strategies to scale up production are numbering-up and sizing-up reactors.^24^ In this paper, we have utilized a numbering-up strategy for scaling up the microwave reactor from lab/pilot scale to industrial scale since no commercial-scale microwave reactor is currently available. This leads to a conservative estimate of the cost of the microwave reactor. Further research in the sizing-up of microwave reactors should lead to a reduction in the cost of the microwave reactor in the future, which will make microwave-enhanced dry reforming even more profitable. Furthermore, external factors such as carbon pricing, tax credits, and decarbonization policies will also be essential in positioning these advanced methods as competitive alternatives to conventional SMR, both environmentally and economically.

## Supplementary Material



## Notes

Stream tables for dry reforming and chemical looping simulations in ASPEN Plus (PDF)

## Notes

This paper was prepared as an account of work partially sponsored by an agency of the United States Government. Neither the United States Government nor any agency thereof, nor any of their employees, makes any warranty, express or implied, or assumes any legal liability or responsibility for the accuracy, completeness, or usefulness of any information, apparatus, product, or process disclosed, or represents that its use would not infringe privately owned rights. Reference herein to any specific commercial product, process, or service by trade name, trademark, manufacturer, or otherwise does not necessarily constitute or imply its endorsement, recommendation, or favoring by the United States Government or any agency thereof. The views and opinions of authors expressed herein do not necessarily state or reflect those of the United States Government or any agency thereof.

## Abbreviations

AEA: Aspen Energy Analyzer
APEA: Aspen Process Economic Analyzer
ATR: auto thermal reforming
CAPEX: capital expenditure
CEPCI: chemical engineering plant cost index
HEN: heat exchange network
HI: heat integration
LCOM: levelized cost of methanol
MILP: mixed-integer linear programming
MTP: million ton per
MTO: methanol to olefin
MW-DRM: microwave-assisted dry reforming of methane
NG: natural gas
NPV: net present value
NRTL: non-random two-liquid
OPEX: operational expenditure
PFD: process flow diagram
POX: partial oxidation
PSA: pressure swing adsorption
RK-SOAVE: Redlich–Kwong–Soave
SMR: steam methane reforming
SN: stoichiometric number
