# Supplementary material for: Technoeconomic Analysis of Microwave-Assisted Dry Reforming Integrated with Chemical Looping for Production of Methanol
Source: Ind Eng Chem Res. 2025 Jun 7;64(24):12074–86. doi: 10.1021/acs.iecr.5c00543 (PMC12186481; doi:10.1021/acs.iecr.5c00543)
Supplement: Supplementary file 1 [file ie5c00543_si_001.pdf]

Technoeconomic Analysis of Microwave-Assisted Dry Reforming Integrated with Chemical Looping for Production of Methanol †

Omar Almaraz[1], Jarrett Riley[2], Srinivas Palanki[1]*

[1]Department of Chemical and Biomedical Engineering

West Virginia University, Morgantown, WV 26506

[2]National Energy Technologies Laboratory

3610 Collins Ferry Road, Morgantown, WV, 25607

Supplementary Data

Table S1: Stream summary for Microwaved Assisted Dry Reforming of Methane Process.

**Stream Name**	**CL-CO2**	**CL-H2**	**CO2**	**METHANOL**	**NG-DRM**	**1**	**2**	**3**	**5**	**6**	**7**
Temperature [C]	25	22	30	69	30	30	30	30	371	375	36
Pressure [bar]	1	25	1	1	1	1	1	1	1	1	1
Molar Vapor Fraction	1	1	1	0.09	1	1	1	1	1	1	1
Mole Flows [kmol/hr]	3537	10100	990	6447	2641	2328	313	2328	2328	2328	2328
				Mole Fractions							
H_2	0.44	99.91	0.00	0.00	0.00	0.00	0.00	0.00	0.00	0.00	0.00
N_2	1.21	0.00	0.00	0.00	0.24	0.24	0.24	0.24	0.24	0.24	0.24
CO	0.34	0.00	0.00	0.00	0.00	0.00	0.00	0.00	0.00	0.00	0.00
CH_4	0.00	0.00	0.00	0.00	94.70	94.70	94.70	94.70	94.70	94.70	94.70
C_2H_6	0.00	0.00	0.00	0.00	3.84	3.84	3.84	3.84	3.84	3.84	3.84
CO_2	96.68	0.00	100.00	0.00	0.25	0.25	0.25	0.25	0.25	0.25	0.25
C_3H_8	0.00	0.00	0.00	0.00	0.82	0.82	0.82	0.82	0.82	0.82	0.82
C_4H_{10}	0.00	0.00	0.00	0.00	0.15	0.15	0.15	0.15	0.15	0.15	0.15
CH_3OH	0.00	0.00	0.00	99.91	0.00	0.00	0.00	0.00	0.00	0.00	0.00
H_2O	1.33	0.09	0.00	0.09	0.00	0.00	0.00	0.00	0.00	0.00	0.00

**Stream Name**	**8**	**9**	**10**	**11**	**12**	**13**	**14**	**15**	**16**	**17**
Temperature [C]	26	29	610	800	800	320	40	40	40	968
Pressure [bar]	1	1	1	1	1	1	1	1	1	76
Molar Vapor Fraction	1	1	1	1	1	1	1	1	1	1
Mole Flows [kmol/hr]	4527	6856	6856	685	11633	11633	11633	11350	22984	22984
					Mole Fractions					
H_2	0.35	0.23	0.23	0.23	40.02	40.02	40.02	100.00	69.64	69.64
N_2	0.95	0.71	0.71	0.71	0.42	0.42	0.42	0.00	0.21	0.21
CO	0.27	0.18	0.18	0.18	41.17	41.17	41.17	0.00	20.84	20.84
CH_4	0.00	32.16	32.16	32.16	0.57	0.57	0.57	0.00	0.29	0.29
C_2H_6	0.00	1.30	1.30	1.30	0.00	0.00	0.00	0.00	0.00	0.00

CO_2	97.41	64.41	64.41	64.41	17.42	17.42	17.42	0.00	8.82	8.82
C_3H_8	0.00	0.28	0.28	0.28	0.00	0.00	0.00	0.00	0.00	0.00
C_4H_{10}	0.00	0.05	0.05	0.05	0.00	0.00	0.00	0.00	0.00	0.00
H_2O	1.04	0.68	0.68	0.68	0.40	0.40	0.40	0.00	0.20	0.20

**Stream Name**	**18**	**19**	**20**	**21**	**22**	**23**	**24**	**25**	**26**	**27**	**28**
Temperature [C]	160	158	188	188	188	254	254	254	221	221	40
Pressure [bar]	76	76	76	76	76	76	76	76	76	71	67
Molar Vapor Fraction	1	1	1	1	1	1	1	1	1	1	0.22
Mole Flows [kmol/hr]	22984	23720	23720	11860	11860	6174	6174	12349	12349	10800	10800
Mole Fractions											
H_2	69.64	70.07	70.07	70.07	70.07	34.87	34.87	34.87	34.87	18.80	18.80
N_2	0.21	0.29	0.29	0.29	0.29	0.56	0.56	0.56	0.56	0.64	0.64
CO	20.84	20.19	20.19	20.19	20.19	0.39	0.39	0.39	0.39	0.00	0.00
CH_4	0.29	0.39	0.39	0.39	0.39	0.75	0.75	0.75	0.75	0.85	0.85
CO_2	8.82	8.84	8.84	8.84	8.84	9.34	9.34	9.34	9.34	3.95	3.95
CH_3OH	0.00	0.02	0.02	0.02	0.02	46.07	46.07	46.07	46.07	59.85	59.85
H_2O	0.20	0.20	0.20	0.20	0.20	8.02	8.02	8.02	8.02	15.90	15.90

**Stream Name**	**29**	**30**	**31**	**32**	**33**	**34**	**35**	**36**	**37**	**38**	**39**
Temperature [C]	40	40	40	40	56	83	40	40	40	40	40
Pressure [bar]	67	67	67	67	77	76	67	67	67	67	67
Molar Vapor Fraction	1	0	1	1	1	1	0.97	1	1	1	1
Mole Flows [kmol/hr]	2413	8386	736	1677	736	736	274	1402	1249	12	140
Mole Fractions											
H_2	83.64	0.15	83.64	83.64	83.64	83.64	0.00	100.00	100.00	100.00	100.00
N_2	2.74	0.03	2.74	2.74	2.74	2.74	16.77	0.00	0.00	0.00	0.00
CH_4	3.53	0.08	3.53	3.53	3.53	3.53	21.57	0.00	0.00	0.00	0.00
CO_2	9.56	2.34	9.56	9.56	9.56	9.56	58.43	0.00	0.00	0.00	0.00

CH_3OH	0.49	76.93	0.49	0.49	0.49	0.49	3.01	0.00	0.00	0.00	0.00
H_2O	0.03	20.47	0.03	0.03	0.03	0.03	0.21	0.00	0.00	0.00	0.00

**Stream Name**	**40**	**41**	**42**	**43**	**44**	**45**	**46**	**47**	**48**	**49**
Temperature [C]	12	12	13	86	87	127	135	69	69	107
Pressure [bar]	6	6	2	2	11	8	8	1	1	1
Molar Vapor Fraction	1	0	1	0	0	0	0	0.20	0	0
Mole Flows [kmol/hr]	50	8336	174	8161	8161	3037	5124	3037	3410	1714
Mole Fractions										
H_2	24.45	0.00	0.11	0.00	0.00	0.00	0.00	0.00	0.00	0.00
N_2	4.22	0.00	0.15	0.00	0.00	0.00	0.00	0.00	0.00	0.00
CH_4	10.62	0.02	0.92	0.00	0.00	0.00	0.00	0.00	0.00	0.00
CO_2	59.68	1.99	95.12	0.00	0.00	0.00	0.00	0.00	0.00	0.00
CH_3OH	0.97	77.39	3.69	78.97	78.97	99.99	66.51	99.99	99.83	0.20
H_2O	0.06	20.59	0.00	21.03	21.03	0.01	33.49	0.01	0.17	99.80

Table S2: Stream summary for Chemical Looping Process.

**Stream Name**	**AIR**	**CL-CO2**	**CL-H2**	**NG-CL**	**NG-HEAT**	**OC**	**STM-SR**	**50**	**51**	**52**	**53**	**54**
Temperature [C]	25	25	22	25	25	970	230	239	775	800	957	871
Pressure [bar]	1	2	25	2	1	2	2	2	2	2	2	2
Molar Vapor Fraction	1	1	1	1	1	0	1	1	1	1	0.04	0.11
Molar Solid Fraction	0	0	0	0	0	1	0	0	0	0	0.96	0.89
Mole Flows [kmol/hr]	133446	3537	10100	3320	825	71297	15158	3320	3320	3320	74239	90312
Mole Fractions												
H_2	0.00	0.44	99.91	0.00	0.00	0.00	0.00	0.00	0.00	0.00	0.00	0.28

CO	0.00	0.34	0.00	0.00	0.00	0.00	0.00	0.00	0.00	0.00	0.00	0.17
N_2	78.99	1.21	0.00	1.29	1.29	0.00	0.00	1.29	1.29	1.29	0.06	0.05
CH_4	0.00	0.00	0.00	93.52	93.52	0.00	0.00	93.52	93.52	93.52	4.18	0.00
CO_2	0.00	96.68	0.00	1.29	1.29	0.00	0.00	1.29	1.29	1.29	0.06	3.63
Fe	0.00	0.00	0.00	0.00	0.00	0.00	0.00	0.00	0.00	0.00	0.00	9.87
H_2O	0.00	1.33	0.09	0.00	0.00	0.00	100.00	0.00	0.00	0.00	0.00	7.05
O_2	21.01	0.00	0.00	0.00	0.00	0.00	0.00	0.00	0.00	0.00	0.00	0.00
$Ca_2Fe_2O_5$	0.00	0.00	0.00	0.00	0.00	0.00	0.00	0.00	0.00	0.00	1.53	0.00
CaO	0.00	0.00	0.00	0.00	0.00	0.00	0.00	0.00	0.00	0.00	0.00	4.94
Fe_3O_4	0.00	0.00	0.00	0.00	0.00	0.00	0.00	0.00	0.00	0.00	1.02	0.00
$CaFe_2O_4$	0.00	0.00	0.00	0.00	0.00	100.00	0.00	0.00	0.00	0.00	92.99	74.01
C_2H_6	0.00	0.00	0.00	3.17	3.17	0.00	0.00	3.17	3.17	3.17	0.14	0.00
C_3H_8	0.00	0.00	0.00	0.69	0.69	0.00	0.00	0.69	0.69	0.69	0.03	0.00
C_4H_{10}	0.00	0.00	0.00	0.04	0.04	0.00	0.00	0.04	0.04	0.04	0.00	0.00

**Stream Name**	**55**	**56**	**57**	**58**	**59**	**60**	**61**	**62**	**63**
Temperature [C]	871	967	960	960	960	722	107	125	25
Pressure [bar]	2	2	2	2	2	2	2	2	2
Molar Vapor Fraction	1	0.12	0.12	0	1	1	0.89	1	0
Molar Solid Fraction	0	0.88	0.88	1	0	0	0	0	0
Mole Flows [kmol/hr]	10099	81396	81018	70919	10099	10099	10099	10099	6561
				Mole Fractions					
H_2	2.52	0.31	0.02	0.00	0.16	0.16	0.16	0.16	0.00
CO	1.50	0.19	0.01	0.00	0.12	0.12	0.12	0.12	0.00
N_2	0.42	0.05	0.05	0.00	0.42	0.42	0.42	0.42	0.00
CO_2	32.49	4.03	4.22	0.00	33.87	33.87	33.87	33.87	0.00
H_2O	63.07	7.83	8.16	0.00	65.43	65.43	65.43	65.43	100.00
$Ca_2Fe_2O_5$	0.00	0.00	1.40	1.60	0.00	0.00	0.00	0.00	0.00
Fe_3O_4	0.00	0.00	0.93	1.06	0.00	0.00	0.00	0.00	0.00
CaFe	0.00	87.59	85.21	97.34	0.00	0.00	0.00	0.00	0.00

Stream Name	65	66	67	68	69	70	71	72	73	74
Temperature [C]	843	874	874	125	125	22	874	877	970	970
Pressure [bar]	2	2	2	2	2	25	2	1	2	2
Molar Vapor Fraction	0.16	0.18	1	1	1	0	0	0.66	0.66	1
Molar Solid Fraction	0.84	0.82	0	0	0	0	1	0.34	0.34	0
Mole Flows [kmol/hr]	95372	85712	15158	15158	12126	2012	70554	207856	206999	135702
Mole Fractions										
H_2	0.00	14.74	83.33	83.33	83.33	0.00	0.00	1.22	0.00	0.00
N_2	0.00	0.00	0.00	0.00	0.00	0.00	0.00	50.72	50.93	77.69
CH_4	0.00	0.00	0.00	0.00	0.00	0.00	0.00	0.37	0.00	0.00
CO_2	0.00	0.00	0.00	0.00	0.00	0.00	0.00	0.01	0.41	0.63
Fe	9.35	0.00	0.00	0.00	0.00	0.00	0.00	0.00	0.00	0.00
H_2O	15.89	2.95	16.67	16.67	16.67	100.00	0.00	0.24	2.26	3.45
O_2	0.00	0.00	0.00	0.00	0.00	0.00	0.00	13.49	11.96	18.24
$Ca_2Fe_2O_5$	0.00	2.60	0.00	0.00	0.00	0.00	3.16	1.07	0.00	0.00
CaO	4.67	0.00	0.00	0.00	0.00	0.00	0.00	0.00	0.00	0.00
Fe_3O_4	0.00	1.73	0.00	0.00	0.00	0.00	2.11	0.71	0.00	0.00
$CaFe_2O_4$	70.08	77.98	0.00	0.00	0.00	0.00	94.73	32.16	34.44	0.00
C_2H_6	0.00	0.00	0.00	0.00	0.00	0.00	0.00	0.01	0.00	0.00

Stream Name	75	76	77	78	79	80	81	82	83
Temperature [C]	240	233	125	99	30	73	150	910	125
Pressure [bar]	2	2	2	2	2	3	3	3	2
Molar Vapor Fraction	1	1	1	1	1	1	1	1	1
Molar Solid Fraction	0	0	0	0	0	0	0	0	0
Mole Flows [kmol/hr]	135702	135702	135702	133446	133446	133446	133446	133446	3032

	Mole Fractions								
H_2	0.00	0.00	0.00	0.00	0.00	0.00	0.00	0.00	83.33
N_2	77.69	77.69	77.69	78.99	78.99	78.99	78.99	78.99	0.00
CO_2	0.63	0.63	0.63	0.00	0.00	0.00	0.00	0.00	0.00
H_2O	3.45	3.45	3.45	0.00	0.00	0.00	0.00	0.00	16.67
O_2	18.24	18.24	18.24	21.01	21.01	21.01	21.01	21.01	0.00
